# Characteristics and outcomes of intensive care readmissions within the first year of lung transplantation: Tertiary care center experience

**DOI:** 10.5339/qmj.2024.qitc.20

**Published:** 2024-03-26

**Authors:** Ahmad Aljumaa, Judy Hejazi, Reem Elmokattaf, Jihad Aljumaa, Abdul Hadi F Afzal, Farooq Pasha

**Affiliations:** Alfaisal University College of Medicine, Riyadh, Saudi Arabia Email: aaljumaa@alfaisal.edu; Emergency Department, King Faisal Specialist Hospital and Research Center, Riyadh, Saudi Arabia

**Keywords:** Lung transplantation, ICU readmission, Outcome, Risk factors, Mortality

## Background

Only a few articles analyze predictive factors in ICU readmission after lung transplantation. This study aims to assess the risk factors, characteristics, and outcomes associated with ICU readmission in lung transplant recipients within the first year.^1–8^

## Research Question

What are the characteristics, risk factors, and outcomes of adult patients who are readmitted to the ICU after lung transplant in a tertiary care hospital in Saudi Arabia?

## Study Design and Methods

This was a retrospective observational study. Electronic medical records of all patients aged 18 years or older who underwent lung transplantation (> 30 days ago) at KFSH&RC and were readmitted to the ICU between January 2013 and January 2022 were reviewed for the following data: demographics, preoperative and intraoperative variables, initial ICU admission, clinical data at readmission, qSOFA score, length of stay, mortality, and use and duration of mechanical ventilation.

## Results

A total of 20 patients were analyzed, and a statistical significance of p < 0.05 was used. The most common indication for transplantation in readmitted patients was COPD, with pneumonia being the most common cause of readmission. Although males accounted for the majority (85%) of readmitted patients, gender was not associated with an increased risk of ICU readmission. Our study reported the lowest rate (11%) of ICU readmissions in the literature, with a mortality rate of 35%. Seventy percent of the patients needed ventilation support, with a mean duration of 9.7 days. In our ICU study, the use of vasopressors and the longer duration of ICU stay were associated with mortality.

## Conclusion

Mortality is high in lung recipients readmitted to the ICU within the first year of transplantation, with pneumonia being the most common cause of readmission. Both the length of ICU stay and the use of a vasopressor have been associated with higher mortality.

## Conflict of Interest

The authors declare no competing interests.
